# Association of Self-Rated Health in Pregnancy With Maternal Childhood Experiences, Socioeconomic Status, Parity, and Choice of Antenatal Care Providers: Cross-Sectional Study

**DOI:** 10.2196/68811

**Published:** 2025-06-03

**Authors:** Bjarne Austad, Gunnhild Åberge Vie, Mari Hegnes Hansen, Hanna Sandbakken Mørkved, Linn Okkenhaug Getz, Bente Prytz Mjølstad

**Affiliations:** 1 General Practice Research Unit, Department of Public Health and Nursing, Faculty of Medicine and Health Sciences, Norwegian University of Science and Technology, P.O. Box 8905, Trondheim, 7491, Norway, 47 99029992

**Keywords:** pregnancy, antenatal care, self-rated health, self-rated mental health, primary care

## Abstract

**Background:**

During pregnancy, self-rated health (SRH) and self-rated mental health (SRMH) are key indicators of health status and predictors of future health care needs. The relationship between pregnant women’s health perceptions and their choice of antenatal care providers, midwives, or general practitioners (GPs) is not known. Factors like childhood experiences and socioeconomic status are important determinants of health throughout life. Understanding these health determinants can help health care providers better address the diverse needs of pregnant women.

**Objective:**

This study aims to assess how SRH and SRMH during pregnancy are associated with maternal childhood experiences, socioeconomic status, parity, and antenatal care provided by midwives or GPs.

**Methods:**

An anonymous, web-based cross-sectional survey was conducted from January to March 2022 among pregnant women in Norway, distributed via Facebook and Instagram. The survey included questions on SRH, SRMH, socioeconomic status, childhood perceptions, and antenatal program participation. Pearson’s chi-squared test and logistic regression models were used to explore associations and estimate odds ratios for good SRH and SRMH.

**Results:**

Among 1402 participants, 94.7% (1328/1402) reported good or very good health before pregnancy, dropping to 67.8% (950/1402) during pregnancy (*P*<.001). Reporting your childhood as good was associated with better SRH compared with those who reported average or difficult childhood (70.2% [755/1076] vs 64% [114/178] vs 53.2% [74/139]; *P*<.001). This corresponds to 48% lower odds of good SRH for those reporting a difficult childhood compared to those reporting a good childhood (OR 0.52, 95% CI 0.36‐0.76). Financial security and higher education were associated with better SRH (both *P*<.001). First-time mothers reported better SRH than those with previous births (73.9% [533/722] vs 61.4% [417/680]; *P*<.001). For SRMH, 89.9% (1260/1402) reported good or very good SRMH before pregnancy, decreasing to 73.1% (1024/1401) during pregnancy (*P*<.001). Women who reported a good childhood, financial security, higher education, and first-time mothers reported better SRMH during pregnancy (*P*<.001 for all). Nearly all women participated in the antenatal program, regardless of their subjective health, and most expressed satisfaction. Among participants, 55.6% (753/1354) received shared antenatal care, 38.6% (520/1354) were seen only by midwives, and 6% (81/1354) only by GPs. The proportion of women receiving antenatal care solely from a midwife decreased with declining SRH, from 42.6% (78/183) among those with very good SRH to 27.3% (15/55) among those with poor SRH.

**Conclusions:**

A difficult maternal childhood, low socioeconomic status, and having given birth before were associated with poorer SRH and SRMH during pregnancy. Both midwives and GPs played vital roles in providing antenatal care, though few women received antenatal care exclusively from GPs. The likelihood of physician involvement in care increased slightly with worsening health.

## Introduction

Throughout pregnancy, women undergo significant physical and psychological changes. Overall health can decline due to common conditions such as nausea, fatigue, and pelvic pain, as well as more serious complications like anemia, gestational diabetes, or preeclampsia. Ensuring maternal health and well-being is a multifaceted challenge that places high demands on health care services and antenatal care [1-3].

Self-rated health (SRH) is a reliable indicator of an individual’s overall health status [4,5] and serves as a strong predictor of future health care needs and mortality [6,7]. In Norway, 9% of individuals assess their health as poor or very poor, consistent with averages observed in other countries from the Organisation for Economic Co-operation and Development (OECD) [8]. Among various factors influencing SRH in adulthood, adverse childhood experiences play a significant role [9-11]. Recent research indicates that maternal adverse childhood experiences can also impact health during pregnancy and contribute to preterm birth [12-14]. In addition, adverse childhood experiences are associated with socioeconomic status [15], which in turn is strongly associated with quality of life during pregnancy [16,17]. A Swedish study found an association between poor SRH during pregnancy and adverse outcomes such as premature birth and small-for-gestational-age newborns [18]. Furthermore, a Brazilian study indicate that primiparous women reported better health compared to multiparous, while another Swedish study noted that women’s physical and emotional SRH is negatively affected by pregnancy but positively influenced by childbirth [19,20]. These findings underscore the importance of assessing pregnant women’s health perceptions to effectively tailor antenatal care.

Poor SRH in pregnancy is associated with elevated rates of depression, anxiety, and stress [21,22]. Prenatal mental health is particularly crucial as it can significantly affect both the mother’s and the baby’s outcomes [23,24]. Mental health issues are prevalent in pregnancy, with an American study estimating that approximately 12% of women experience a major depressive episode during pregnancy [25,26]. In Norway, over 9% of women report experiencing a postpartum depression [27]. While some studies suggest that mental health may remain stable or even improve during pregnancy [16], women with low social support are at an increased risk of developing mental health challenges [28]. Therefore, health care professionals are encouraged to address psychosocial stressors, enhance social support, and evaluate self-rated mental health (SRMH) within antenatal care [24,29].

Antenatal care serves a preventive health service designed to ensure safe pregnancies and childbirth while effectively identifying and managing health risks [30]. In Norway, a comprehensive free antenatal program is available, which includes 9 routine check-ups and early ultrasound examinations during weeks 11‐13 as well as standard ultrasounds at weeks 17‐19 (Textbox 1). National guidelines recommend addressing mental health, although SRH is not specifically mentioned [31]. Norway has one of the lowest infant mortality rates globally and a low rate of maternal mortality, indicating a healthy population and high-quality antenatal care [32-34].

The Regular GP Scheme in Norway ensures that all residents have access to a personal general practitioner (GP), who is expected to provide antenatal care. In addition, midwives in municipalities offer antenatal services. A strong emphasis is placed on the preferences of pregnant individuals, allowing them to choose between midwife- or GP-led check-ups, or a combination of both [31]. However, the limited availability of GPs and midwives may restrict these choices. Notably, pregnant women in Norway have historically attended more antenatal visits than the national guidelines recommend (12 visits instead of the advised 8‐9) [35,36], and over 60% report taking sick leave during pregnancy [37]. Unlike physicians, midwives in Norway are not authorized to certify sick leaves but can issue “maternity allowance” if a mother’s work poses risks to the fetus [38].

Textbox 1. **Characteristics of current Norwegian antenatal care as described in the national guidelines.**

- All pregnant women living in Norway are entitled to free antenatal care.
- The routine program is standardized for all women, regardless of previous childbirth experience, and consists of 9 checkups.
- The first visit is recommended between weeks 6-8, as early as possible, to address lifestyle factors.
- The routine antenatal care program includes ultrasound scans during week 11-13 (gradually implemented since 2022) and between weeks 17-19 (established in the early 1990s).
- Antenatal care is delivered in primary care settings by midwives and regular general practitioners (GPs). Most women in Norway have a designated regular GP and can choose to receive antenatal consultations from their GP, a midwife, or both.
- For high-risk or complicated pregnancies, referrals to specialized health care (gynecologists) are made, although some follow-up care still occurs in primary care settings.

Despite the importance of antenatal care as a preventive health service, details on antenatal care across different health care providers are not readily identified in Norwegian national registries. Updated data on pregnant women’s perceived health and how this impacts their choice of antenatal care providers, midwives, or GPs can aid in the organization of services. Identifying social risk factors associated with poor perceived health in pregnancy can help target preventive efforts, thus mitigating a potential intergenerational impact.

The aim of this study was to examine how SRH and SRMH in pregnancy are associated with (1) maternal childhood experiences, current socioeconomic status, and parity, and (2) antenatal care provided by midwives or GPs.

## Methods

### Study Design and Setting

We conducted a web-based cross-sectional survey among Norwegian pregnant women aged 18 years and older between January and March 2022. The survey was distributed on 6 Facebook (Meta Platforms) groups for pregnant women, organized by the due date, and 5 Instagram (Meta Platforms) accounts focused on pregnancy and childbirth. These groups and accounts had a combined total of 109,000 followers although many women were likely active on multiple platforms. For comparison, 51,292 women gave birth in Norway in 2022.

To promote the survey, we used an introduction video and postings. After publishing the survey on a new platform, all responses were received within 4 days. Data collection was closed after 2‐4 weeks for the different groups as we received no further responses. Of the 1402 women who answered the survey; 868 responses came from Facebook and 534 from Instagram. All participants consented to participate by answering the questionnaire, which included an information letter about the study. The study was anonymous, and the women were asked to respond only once. We followed the Checklist for Reporting Results of Internet E-Surveys (CHERRIES), a quality checklist for internet-based surveys [39].

### Questionnaire

In the absence of validated questionnaires that fully met the study’s broader objectives, we developed a new one. We incorporated validated questions from previous surveys, including questions from the Cambridge Worry Scale and questions related to demographic variables, SRH and childhood experiences from the HUNT study [40,41]. The question “worries about money problems” in the Cambridge Worry Scale seemed somewhat unfamiliar within the context of the Norwegian welfare system. We chose to ask about capacity to manage an unexpected expense as this is easy to understand and has previously been used in Norwegian surveys [42]. In addition, the capacity to manage unexpected expenses of varying amounts has been defined as financial security; we chose NOK 10,000 (approximately US $1000) [43,44]. A total of 10 pregnant women were invited to pilot the survey for assessing completion time and question clarity, of whom 6 responded, suggesting minor linguistic adjustments. The questionnaire was administered by using a data collection tool (Nettskjema.no developed by the University of Oslo), which is user-friendly and compatible with various digital devices. The questionnaire contained up to 57 questions depending on the respondent’s answers, covering five different topics: (1) demographic variables, including sick leave and financial security; (2) assessment of SRH and SRMH, both during the current pregnancy and before pregnancy; (3) antenatal care, including visits to midwives, GPs, gynecologists, ultrasounds, and satisfaction with the controls; (4) sources where the women seek advice and information during pregnancy; (5) thoughts about the time after birth and the parental role, including assessment of own childhood. In total, 12 questions were open-ended with free text boxes, while the rest were closed-ended items. The complete questionnaire is translated into English and attached in Multimedia Appendix 1. This paper examines the parts related to health and attendance in the antenatal care program. Other parts of the project are published separately [45].

### Study Variables

The questions concerning SRH were “Before you became pregnant, how would you characterize your own health?” and “How is your health now?” For SRMH, we asked, “Before you became pregnant, how were you feeling emotionally/mentally?” and “How are you feeling emotionally/mentally now during the pregnancy?” The answer options were very good, good, not so good, or poor in line with other studies [18,19], and were dichotomized for the logistic regression [46]. Participants were asked if they had been or were still on sick leave (used in cases of maternal illness) or maternity allowance (used when mother’s work could be harmful to the fetus). The questions regarding the socioeconomic status included the level of completed education and finances. We did not inquire about income. Instead, participants were asked if they could manage an unforeseen expense of NOK 10,000 (approximately 1000 USD), with response options of yes, uncertain, or no. We merged uncertain and no and defined that as “financial insecurity,” and yes as “financial security.” Furthermore, they were asked a single-item question to describe their childhood as either very good, good, average, difficult, or very difficult. We merged very good and good into good and difficult and very difficult into difficult. Antenatal visits were defined as the regular controls according to the Norwegian antenatal program. The women were asked if they had attended all of them, most of them, not attended, or not relevant (not been to the first control yet) with possible free-text answers for reasons for not attending. In addition, they were asked about the profession of the health care provider (GP, midwife, gynecologist, and others).

### Statistical Analysis

Data were analyzed using SPSS Statistics. The Pearson chi-squared test was used to examine associations between SRH and SRMH, and childhood experiences, financial security, parity, and attendance to antenatal care providers (GP or midwife). We further estimated the odds ratio (OR) of good SRH and SRMH using logistic regression models, and present crude associations as well as associations adjusted for age, education and trimester. While *χ*^2^ tests allowed comparison of all four levels of SRH and SRMH, logistic regression allowed adjustment for potential confounders after dichotomizing perceived health. Most respondents answered all questions; data on trimester was missing for 2 participants, financial security for 2 and childhood experiences for 9 participants. All participants reported SRH and all but 1 SRMH. We performed complete case analyses and excluded those with missing responses from each analysis. Free text responses were analyzed for content and categorized.

### Ethical Considerations

A remit assessment was submitted to the Regional Committee for Medical Research Ethics in Central Norway (REK), which determined that formal ethics approval was not required under national regulations (reference 267956). The study was also approved by the Norwegian Centre for Research Data (NSD; reference 134424), which confirmed that no direct or indirect personal identifiers were processed. All procedures involving human participants were conducted in accordance with relevant national and institutional guidelines and regulations. An information letter, empathetically worded and based on a template provided by NSD, was distributed to all participants. Informed consent was obtained from all participants prior to data collection. No compensation was provided for participation.

## Results

Table 1 shows the characteristics of the study population of 1402 participants. In Multimedia Appendix 2, we have compared these with the national birth statistics for Norway in the year of the survey, 2022.

**Table 1.** Demographic characteristics of the study population of pregnant women in Norway (N=1393‐1402).

Characteristic	n (%)	
Age (years)		
<25	119 (8.5)	
25‐37	1226 (87.4)	
>37	57 (4.1)	
Parity		
First-time mothers	722 (51.5)	
Previous birth	680 (48.5)	
Current trimester		
First trimester	99 (7.1)	
Second trimester	776 (55.4)	
Third trimester	525 (37.5)	
Education		
Lower secondary school	28 (2)	
Upper secondary school	315 (22.5)	
College or university (less than 4 years)	495 (35.3)	
College or university (4 years or more)	564 (40.2)	
Employment status		
Employed	1210 (86.3)	
Student or apprentice	123 (8.8)	
Unemployed (looking for a job)	12 (0.9)	
On welfare benefits, unable to work	44 (3.1)	
Housewife with housework or caring responsibilities	13 (0.9)	
Sick leave during pregnancy		
Yes	820 (58.6)	
No	580 (41.4)	
Maternity allowance^a^		
Yes	197 (14.2)	
No	1195 (85.8)	
Ability to handle unforeseeable expenses of NOK 10,000 (US $1000)		
Yes	1166 (83.3)	
No or do not know	234 (16.7)	
Childhood		
Good or very good	1076 (76.7)	
Average	178 (12.7)	
Difficult or very difficult	139 (9.9)	
Have you attended all your antenatal care visits?		
Yes, all	1323 (94.4)	
Yes, most of them	35 (2.5)	
No	3 (0.2)	
Not relevant or have not been to the first control yet	40 (2.9)	
Who has performed your antenatal care visits thus far?^b^		
Only GP^c^	81 (6)	
Only midwife	520 (38.4)	
GP and midwife	753 (55.6)	
Women in third trimester: Who has performed your antenatal care visits thus far?		
Only GP	15 (2.9)	
Only midwife	240 (45.8)	
GP and midwife	269 (51.3)	
Neither midwife nor GP	1 (0.2)	
Have you had an antenatal visit by a gynecologist in addition to GP or midwife?		
Yes	177 (12.6)	
No	1225 (87.4)	
Overall, how satisfied are you with your antenatal care visits?		
Large or very large extent	1079 (77.1)	
Some or small extent	273 (19.5)	
Not at all	9 (0.6)	
Not relevant or have not been to the first control yet	39 (2.8)	
Have you attended an early ultrasound (before week 17)?		
Yes	1247 (89)	
No	154 (11)	
Have you attended routine ultrasound (weeks 17‐19)?^d^		
Yes	521 (99.4)	
No	3 (0.6)	

a Maternity allowance is an economic compensation to healthy pregnant women who are unable to work because their work may be harmful for the fetus.

b Participants with no controls by GP or midwife thus far in pregnancy were excluded (n=48).

c GP: general practitioner.

d Based on women in 3rd trimester.

### Overall Self-Rated Health Before and During Pregnancy

Before pregnancy, 94.7% (1328/1402) of participants reported their health as either very good (35.2%, 494/1402)) or good (59.5%, 834/1402). During pregnancy, this combined percentage dropped to 67.8% (950/1402) (very good 13.7% (192/1402) and good 54.1% (758/1402). Conversely, the proportion of 5.3% (74/1402) reporting their health as either not so good (4.9%, 68/1402) or poor (0.4%, 6/1402) before pregnancy increased to 32.2% (452/1402) (not so good 28.2% (396/1402) and poor 4% (56/1402) during pregnancy. This indicates a significant decline in SRH (*χ*²_9_=564.7; *P*<.001).

**Figure 1.**
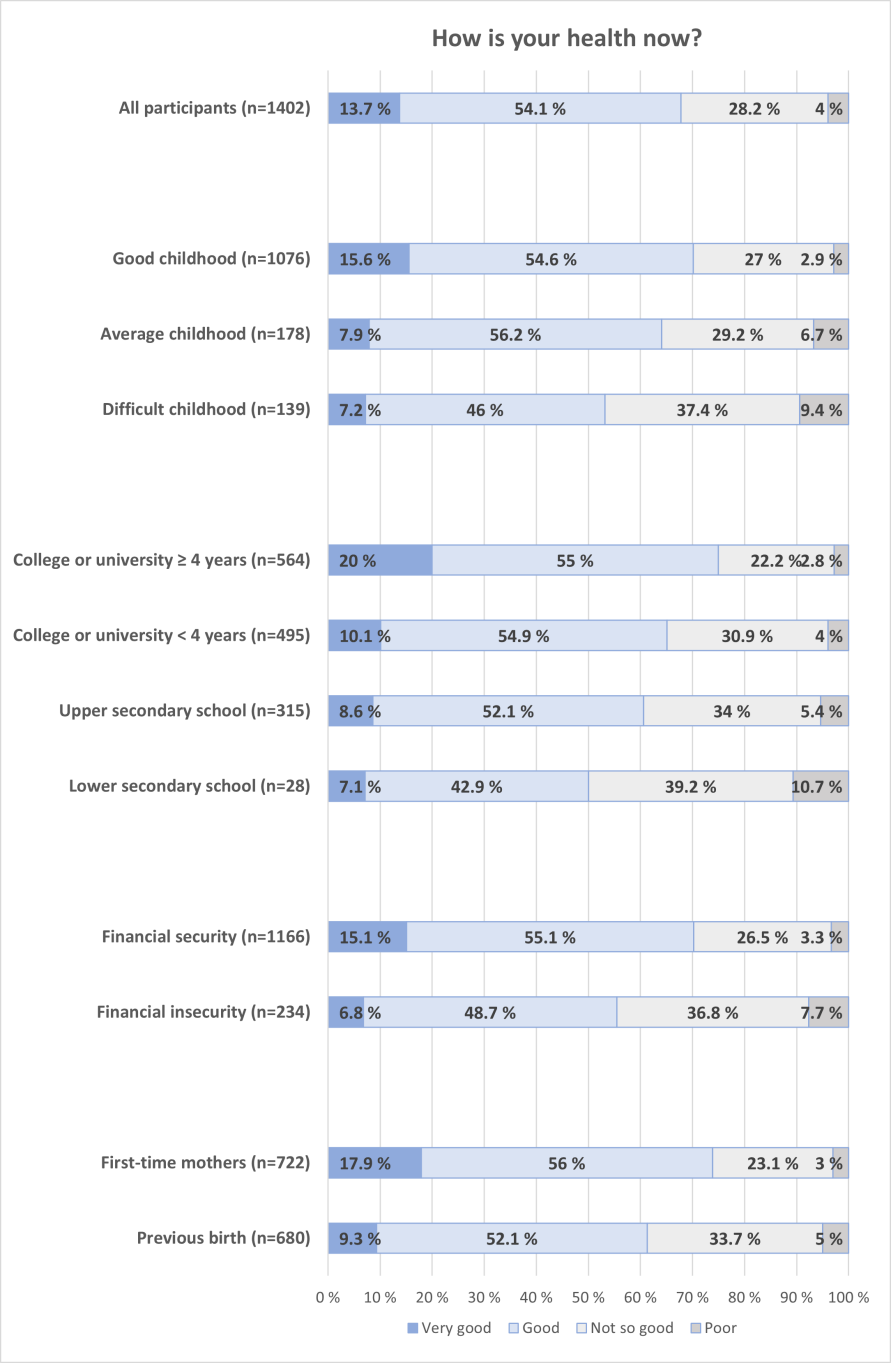
Overall self-rated health during pregnancy distributed by reported childhood, completed education, financial security, and parity. Financial security: the ability to handle unforeseeable expenses of NOK 10,000 (US $1000); n=1400‐1402.

In Figure 1, we present the distribution of overall SRH during pregnancy, categorized by childhood experiences, socioeconomic factors (education and financial status), and parity. In Multimedia Appendix 3, we present crude and adjusted OR for good versus poor SRH. Participants who reported a good childhood had better SRH compared to those with average or difficult childhoods (70.2% [755/1076] vs 64% [114/178] vs 53.2% [74/139]; *χ*²_6_=34.7; *P*<.001). This corresponds to 52% lower odds of good SRH for those with a difficult childhood (OR 0.48, 95% CI 0.34‐0.69), and the difference was robust to adjustment for age, education, and trimester (adjusted OR 0.52, 95% CI 0.36‐0.76; see Multimedia Appendix 3).

Women with financial security reported better SRH than those with financial insecurity (70.2% [819/1166] vs 55.6% [130/234]; *P*<.001), corresponding to 36% lower odds of good SRH for women with financial insecurity in the adjusted model (OR 0.64, 95% CI 0.47‐0.87). Higher education was also associated with better SRH (*χ*²_9_=49.3; *P*<.001), with 76% lower odds for good SRH for those with only lower secondary school compared to 4 years or more of college or university (adjusted OR 0.33, 95% CI 0.16‐0.72). First-time mothers reported better SRH than mothers with previous births (73.9% [533/722] vs 61.4% [417/680]; *P*<.001), with 47% lower odds of good SRH for women with previous births (adjusted OR 0.53, 95% CI 0.41‐0.67).

### Self-Rated Mental Health Before and During Pregnancy

Before pregnancy, 89.9% (1260/1402) of participants reported their SRMH as very good (33.8%, 474/1402) or good (56.1%, 786/1402). During pregnancy, this combined percentage dropped to 73.1% (1024/1401; very good 17.1% [239/1401] and good 56% [785/1401]). Conversely, while 10.2% (142/1402) reporting their SRMH as either not so good (9.3%, 130/1402) or poor (0.9%, 12/1402) before pregnancy, this increased to 26.9% (377/1401; not so good 23.1%, 323/1401) and poor (3.9%, 54/1401) during pregnancy. This indicates a significant decline in SRMH (*χ*²_9_=365; *P*<.001).

**Figure 2.**
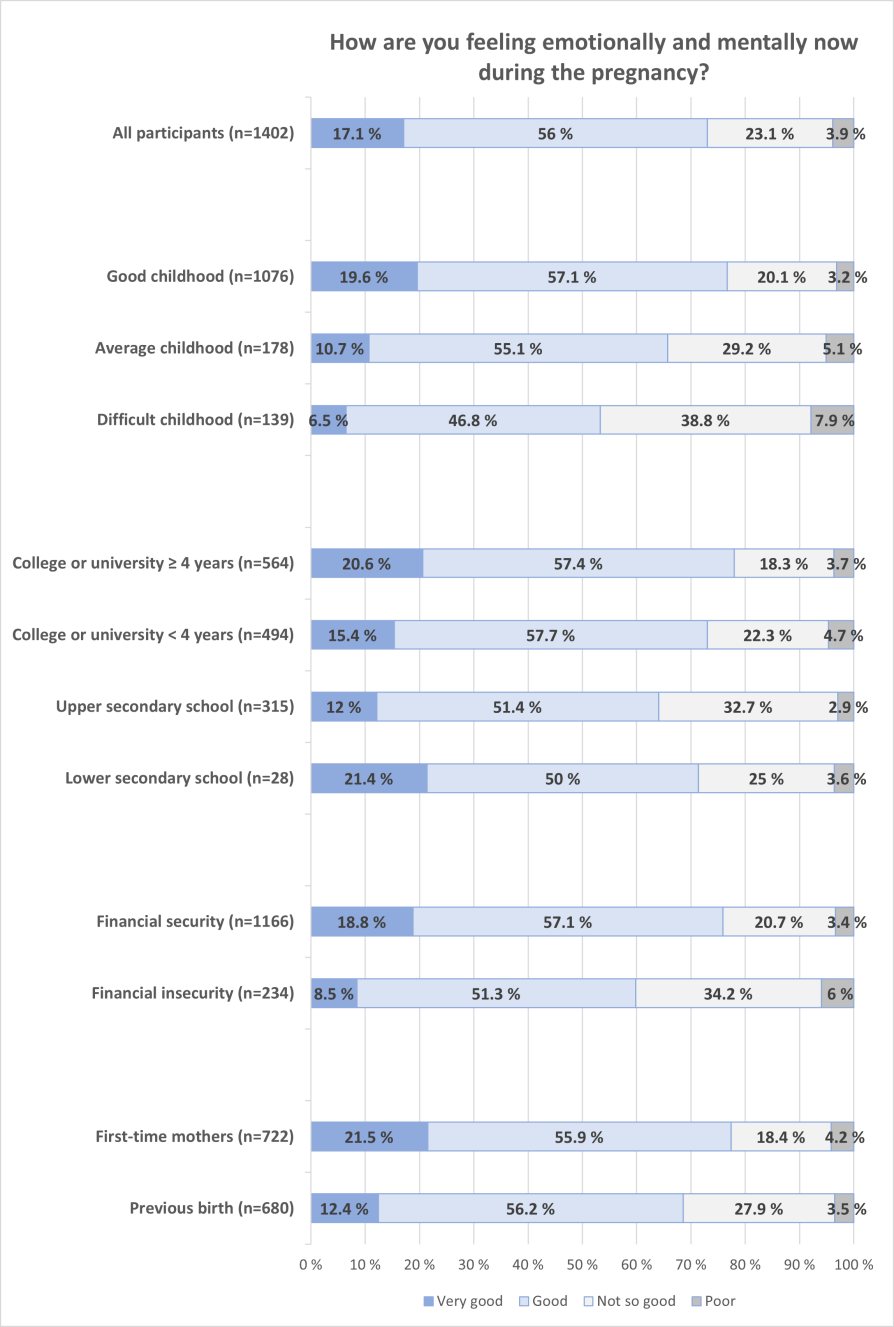
Self-rated mental health during pregnancy distributed by reported childhood, completed education, financial security, and parity. Financial security: the ability to handle unforeseeable expenses of NOK 10,000 (US $1000); n=1400‐1402.

Figure 2 shows the distribution of SRMH during pregnancy by childhood experiences, socioeconomic factors (education and financial status), and parity. Multimedia Appendix 3 shows crude and adjusted ORs for good SRMH. Participants who described their childhood as good reported better SRMH compared to those with average or difficult childhoods (76.7% [825/1075] vs 65.7% [117/178] vs 53.3% [65/139]; *χ*²_6_=49.7; *P*<.001). This corresponds to 65% lower odds of good SRMH for those with a difficult childhood (OR 0.35, 95% CI 0.24‐0.56), and the difference was robust to adjustment for age, education, and trimester (adjusted OR 0.37, 95% CI 0.25‐0.54; see Multimedia Appendix 4).

The proportion of women reporting good or very good SRMH increased with longer education, except for the few (n=28) with only lower secondary school (78% [440/564] vs 73.1% [361/494] vs 64.4% [203/315] vs 71.4% [20/28]; *χ*²_9_=30.2; *P*<.001). The odds of good SRMH were 45% lower for those with upper secondary school compared to those with more than 4 years of college or university (adjusted OR 0.55, 95% CI 0.40‐0.76). Women with financial security reported better SRMH than those with financial insecurity (75.9% [884/1402] vs 59.8% [140/234]; *P*<.001), corresponding to 46% lower odds for good SRMH for those with financial insecurity (adjusted OR 0.54, 95% CI 0.39‐0.74). First-time mothers reported better SRMH than mothers with previous births (77.4% [558/721] vs 68.5% [466/680]; *P*<.001), with 43% lower odds for good SRMH for women with previous births (adjusted OR 0.57, 95% CI 0.45‐0.73).

### Sick Leave and Maternity Allowance

In total, 58.6% (820/1400) reported that they were or had been on sick leave so far during pregnancy (see Table 1), cumulating to 69.8% (366/525) among women in the third trimester (not shown in the table). Sick leave was strongly associated with SRH: 29.8% (57/191) of those reporting very good health, 53% (401/757) with good health, 80.6% (319/396) with not so good health, and 76.8% (43/56) with poor health were or had been on sick leave (*P*<.001). A similar pattern was observed for SRMH: 44.4% (106/239) of those with very good SRMH, 56.4% (442/784) with good SRMH, 70.5% (227/322) with not so good SRMH, and 83.3% (45/54) with poor SRMH were or had been on sick leave (*P*<.001). The proportion receiving maternity allowance remained stable at approximately 14% (197/1392; see Table 1) and was not affected by SRH (*P*=.99) or SRMH (*P*=.68).

### Attendance in the Antenatal Program and Satisfaction

Since nearly all attended the antenatal program (see Table 1), there was no numerical basis for analyzing whether the attenders had any different SRH than the nonattenders. 24 participants (1.7%) responded to an open-ended question about reasons for not attending all antenatal checkups thus far. The reasons for missing appointments were postponing due to illness (either their own or the health care personnel’s, such as COVID-19; n=9), being early in pregnancy and not yet needing a check-up (n=4), being advised by their health care provider to wait or experiencing a wait time for the first appointment (n=4), delaying due to vacation or other reasons (n=3), forgetting the appointment (n=2), or not needing a check-up because they received treatment at the hospital (n=2).

In total, 55.6% (753/1354) received shared care from both their GP and midwife, 38.4% (520/1354) had only been to a midwife and 6% (81/1354) to only their GP. The 13 % (177/1357) that had seen a gynecologist during pregnancy were mostly also seen by either a GP or midwife, and only 0.2% (3/1357) reported having solely been to a gynecologist (not shown in the table). We did a separately analyze for women in the 3rd trimester as they probably have been to most antenatal visits and found an even lower proportion women only seeing their GP (2.9%, 15/525) and only 0.2% (1/525) responded to neither have seen a GP nor midwife.

On a 5-point Likert scale, most participants were satisfied to a large or very large extent with the antenatal care visits (79.3%, 1079/1361). While the satisfaction levels were slightly lower for those who received antenatal visits solely from their GPs compared to those who had seemed midwife only or shared care, this difference was not statistically significant (70.4% [57/81] vs 81.7% [424/519] vs 78.9% [593/752]; *P*=.05). In addition, there were no notable differences in the proportion of participants reporting being satisfied to a little extent or not at all (3.7% [3/81] vs 3.5% [18/519] vs 3.6% [27/752]).

### Self-Rated Health and Attendance

**Figure 3.**
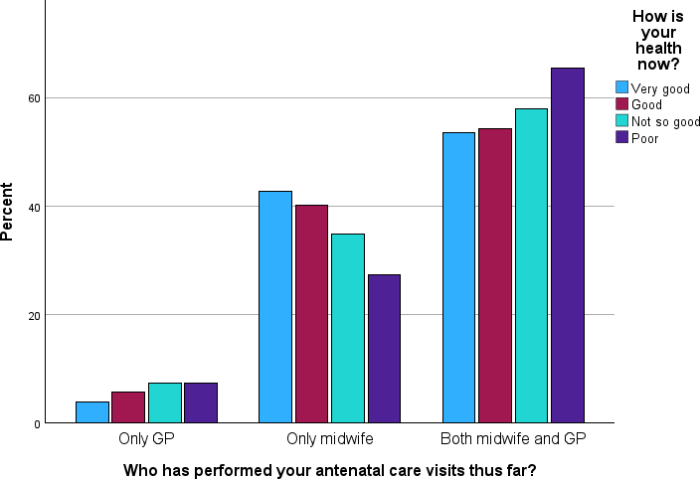
Self-rated health during pregnancy for participants having antenatal consultation with only a general practitioner (GP; n=81), only a midwife (n=520) or combined GP and midwife (n=753). Participants with no controls by GP or midwife thus far in pregnancy were excluded (n=48).

**Figure 4.**
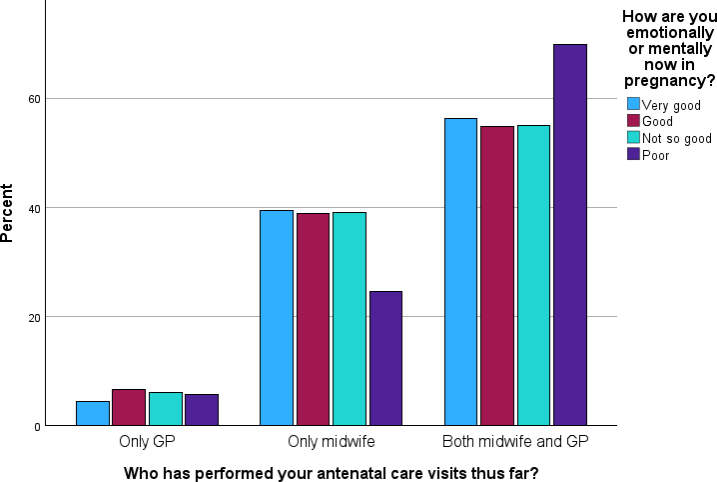
Self-rated mental health during pregnancy for participants having antenatal consultation with only a general practitioner (GP; n=81), only a midwife (n=520) or combined GP and midwife (n=753). Participants with no controls by GP or midwife thus far in pregnancy were excluded (n=48).

We analyzed the distribution of antenatal care visits based on SRH (see Figure 3) and SRMH (see Figure 4). The proportion of women receiving antenatal care solely from a midwife decreased as SRH declined, from 42.6% (78/183) among those with very good SRH to 27.3% (15/55) among those with poor SRH. The proportion receiving combined care increased correspondingly with poorer SRH, while the proportion receiving antenatal care solely from a GP remained low. These differences were not statistically significant across all 3 categories of attendance (*χ*²_6_=9.1, *P*=.17). However, we additionally compared visits to GPs, either solely or as shared care, to visits solely to midwives, with results indicating a statistically significant difference (*χ*²_3_=8.1; *P*=.04). We also examined antenatal visits by gynecologists, whether solely or in combination with primary health care, and found an increase from 13% among women with very good SRH to 23.2% with poor SRH (*P*<.001; not shown in Figure 3).

The proportion of women receiving antenatal care solely from a midwife remained stable for those with very good (39.3%, 90/129), good (38.8%, 292/412), and not so good (39%, 124/175) SRMH, but dropped for those reporting poor SRMH (24.5%, 13/53). However, this difference was not statistically significant, whether assessed across all 3 categories of attendance (*χ*²_6_=6.2; *P*=.40) or comparing any visits to GPs to visits solely to midwives (*χ*²_3_=5.2; *P*=.16). For gynecologists, there were no statistically significant differences by different SRMHs (*P*=.14; not shown in Figure 4).

## Discussion

### Principal Findings

We found that both SRH and SRMH were reported to be worse during pregnancy compared to before pregnancy. Women who had experienced difficult childhoods, had low socioeconomic status, or those who have given birth before reported poorer SRH and SRMH, even after adjusting for age, trimester, and education. Nearly all pregnant women participated in the antenatal program regardless of their health status. Both midwives and GPs played crucial roles in providing antenatal care, shared care being the most common type of care. Few women received antenatal visits exclusively from a GP. Notably, the proportion of women receiving antenatal care from a physician (either GP or gynecologist) increased slightly with declining SRH.

### Findings in Relation to Comparable Studies

#### Self-Rated Health Before and During Pregnancy

While it is well established that health can temporarily deteriorate during pregnancy [47,48], the extent of worsening health reported by the women in our study is striking. The proportion of women indicating their SRH as not so good or poor, raised from 5.3% (74/1402) to 32.2% (452/1402), and their SRMH from 10.2% (142/1402) to 26.9% (377/1402). Direct comparisons with other studies are complicated by variations in exact phrasing and participant selection, in addition to sociocultural differences across countries [49]. A Swedish register study noted an increase in poor SRH from approximately 3% before pregnancy to 9% during pregnancy [50], while an American study found that 16% of women indicated fair or poor SRH during pregnancy [5]. In contrast, another Swedish study reported similar findings to ours, with the proportion of women rating their SRH as poor increasing from 20% to 37% between mid- and late pregnancy [19]. In the Norwegian context, our survey question—“Before you became pregnant, how would you characterize your own health?”—yielded a proportion of poor SRH that aligns with or is slightly lower than national averages regardless of pregnancy status [8]. We collected data shortly after the lifting of COVID-19 restrictions in Norway, and it remains unclear whether some participants’ reporting of their health before or during pregnancy were still negatively impacted by the pandemic [51,52].

Adverse childhood experiences are associated with poor health in adult life [9-11,53] and epigenetic aging later in life, including during pregnancy [12]. Furthermore, these experiences are associated with an increased risk of preterm birth and hypertensive disorders in pregnancy [14,54]. A recent study highlighted links between such experiences and risk factors among pregnant women, including smoking, depression, and being exposed to physical abuse, all of which can adversely impact maternal, pregnancy and infant health [55]. A Danish study found that adverse childhood experiences was associated with antenatal depression and anxiety, however, the association was not as strong as that of recent stressful life events [56]. Our findings, which indicate 52% lower odds of good SRH and 65% lower odds of good SRMH if they perceived their childhood as difficult, align well with these studies. Although we did not explore various categories of adverse childhood experiences, the single question regarding women’s assessment of their childhood is validated and represents an empirically supported method for evaluating such experiences [57]. This underscores the importance of addressing maternal childhood experiences during antenatal visits in relation to SRH and SRMH [18].

Improved socioeconomic status is well-documented to correlate with better health outcomes, influencing both pregnancy and the likelihood of complications, possibly linked to increased stress [56,58,59]. A global analysis emphasized how social determinants can affect maternal health in a complex interplay between superdeterminants, individual factors and health care systems [3]. Our findings indicate that higher education and financial security is associated with better SRH and SRMH. This is particularly noteworthy given that Norway has one of the world’s most comprehensive welfare systems and robust birth rights, including up to 12 months of paid parental leave and free antenatal care. In another study using the same dataset, we found that 11.8% (165/1402) of the participants were greatly concerned about their financial situation, and nearly 3 of 4 worried to some extent [45]. As a comparison, in a study of pregnant and postpartum women in the United States, 59% reported health care unaffordability and 54% general financial stress [60].

#### Sick Leave and Maternity Allowance

International comparisons of sick leave prevalence are challenging due to differences in welfare schemes and labor markets; however, Norway reports some of the highest rates in Europe [37]. Previous studies indicate that between 60% and 75% of pregnant women in Norway report sick leave during pregnancy [37,61]. This aligns with our findings, where nearly 60% (820/1,402) reported at least one instance of sick leave, increasing to 70% (366/525) among women in the third trimester. The reasons for frequent sick leave are complex [62]. Although an appropriate level of sick leave is difficult to establish, the observed increase in sick leave among those with poorer SRH or SMRH aligns well with the intention of its use. In addition, the finding that maternity allowance [38]—granted when a mother’s work may be harmful to the fetus—was not influenced by the women’s health further supports the rationale behind its use.

#### Antenatal Care Provision by Midwife, GP, or Both?

While other studies have found several barriers to antenatal care, our findings indicate that nearly all pregnant women participated in the antenatal care program, irrespective of their health status [63]. Shared care was the most common type of antenatal care (55.6%), compared to only seeing a midwife (38.4%) or a GP (6%). According to the Norwegian Health Atlas, based on registry data, midwives handled 57% of antenatal contacts in primary care, while GPs or out-of-hours accounted for 43%. However, the proportion receiving shared care was not accounted for [36]. A Norwegian non–peer-reviewed survey among pregnant women and parents of young children reported that two-thirds of women received shared antenatal care between midwives and GPs [64].

Most women were satisfied with the antenatal care. Our study found lower involvement of GPs compared to other studies, which was unexpected. Several factors could contribute to the higher attendance of pregnant women at midwifery services. A possible explanation could be the limited availability of GPs due to high workloads and a growing number of unfilled positions. By September 2022, approximately 4% of Norwegians were without a GP [65]. While midwifery services in municipalities have been significantly strengthened, with a nearly 70% increase from 2015 to 2020 [66], the number of GPs grew by less than 8% over the same period [67]. Another reason for the higher attendance at midwives’ services could be the longer consultations and their specialized training and experience in antenatal care. This discrepancy is worth debating, as midwives are also a limited resource at a time when Norway, like many other countries, needs to focus on prioritization and sustainability in health services to secure good antenatal care for all, including those who need it the most [68].

A key goal of antenatal care is to identify individual vulnerability factors that indicate an increased risk of complications during pregnancy, childbirth, or the postpartum period [69-71]. Midwives can provide valuable continuity of care throughout pregnancy, which has been shown to be beneficial [72,73]. A GP working within a continuity-of-care care model is ideally positioned to assess vulnerability, drawing on their comprehensive knowledge of both the woman’s psychosocial circumstances and prior medical history [74,75]. Furthermore, since newborns are typically registered with the same GP as their mother, both mother and child are likely to benefit from the regular GP’s unique life course perspective [76]. A registry study found that an increase in GP density in Norway significantly improved perinatal outcomes, including reduced fetal deaths and higher birth weights [77]. Although our study was not designed to assess complications, our finding that poor SRH was linked to more frequent contact with physicians (GPs or gynecologists) supports this argument. We do not know the reason for the more frequent contact, but the Norwegian antenatal guideline recommends seeking a physician for certain conditions, such as serious health issues, contagious diseases, and serious symptoms like bleeding and strong pain [31].

While pregnant women in Norway can chose between midwife- or GP-led checkups, the antenatal program in Denmark is designed for shared care. It includes three GP assessments (at gestational weeks 6‐10, 25, and 32) combined with five to seven appointments with a midwife [78]. The fact that most participants in our study received shared antenatal care from both GPs and midwives indicates that Norwegian women might prefer this kind of care [64]. However, the coordination of antenatal visits between midwives and GPs is often left to the woman herself, and this could be a reason for the established habits of attending more antenatal checkups than formally recommended in national guidelines [36]. Therefore, there are compelling arguments for formalizing shared care and securing GP involvement in pregnancy care in Norway, also from a sustainability perspective. Further research is needed for this topic, especially concerning pregnant women with poorer health. The key challenge will be to establish effective communication among all involved professionals as interdisciplinary collaboration has been found to be complex [74,79]. Although electronic messages between health personnel is possible in Norway, continuity of information flow in pregnancy care is currently secured by the woman carrying a physical “Antenatal Card” [80]. An ongoing national digitalization project is, however, expected to facillitate a seamless electronic communication in the future.

### Methodological Strengths and Limitations

We consider the face validity of the questionnaire to be strong. This is supported by a successful pilot test with minimal feedback, and very few missing data, suggesting that participants found the study both relevant and well-aligned with its objectives. Although the authors developed the questionnaire, it contained several validated questions [41]. The questionnaire was anonymous, and participants had the opportunity to answer multiple times, as the data collection tool (Nettskjema.no) did not track IP addresses. Members of overlapping social media groups would receive an invitation for each group they belonged to. Although we considered this before the study began, we concluded that multiple responses from the same participant were unlikely, among other reasons because the survey was quite comprehensive. Furthermore, receiving all answers within few days lowers the risk of multiple responses. However, we cannot completely rule it out. Missing data was negligible and would not have substantially changed results, even if the missing data were not at random. Therefore, more advanced methods like multiple imputation were not considered. We collected data shortly after COVID-19 restrictions were lifted in Norway, and its possible that participants’ health and their recall of their health before pregnancy was still adversely affected by the pandemic [51,52].

Although we received 1402 responses, which is substantial, it represents only a portion of the 51,292 women who gave birth in Norway in 2022. Therefore, while we recognize the significance of our findings, we must remain cautious about the weight we assign to the results. Odds ratios are greater than risk ratios when the outcome is frequent and should not be read as the relative risk. The sociodemographic characteristics of the study population (age, parity, and marital status) were comparable to the general population of pregnant women in Norway, except for a lower proportion of immigrants. Findings therefore might not be generalizable to immigrants. Self-selection to participation might also depend on the included topics, such as worries and strategies for information seeking. The broad age categories increase the chances of residual confounding by age. Beyond these sociodemographic factors, it is challenging to evaluate the representativity of SRH and SRMH among our participants. Since SRH and SRMH were assessed retrospectively, recall bias could be a concern. However, it may have only been a few months since the participants were not pregnant. In addition, we expect recall of such broad concepts to be reasonably accurate, and our findings of their SRH align closely with average SRH reported in national statistics [8].

We used single-item measures for SRH to describe participants’ health. Although this subjective measure might seem simplistic and some nuances in variations in health may be lost, it is a comprehensive indicator that has been shown to correlate well with morbidity and mortality [4,81]. Furthermore, single-item SRMH correlates moderately with mental health scales and appears to be a robust measure of population mental health [82]. However, SRMH is not precisely defined, and assessment using the word “emotionally” in addition to “mentally” may have lowered the threshold for reporting poor SRMH, but overall, these single-item measures remain valuable tools for understanding the health status of our participants. Still, to get more comprehensive insights, future studies may consider incorporating multi-item scales.

### Conclusion

Both SRH and SRMH were reported to be worse during pregnancy compared to before. Factors such as a difficult maternal childhood, low socioeconomic status, and previous childbirth were associated with poorer health during pregnancy. Both midwives and GPs played crucial roles in delivering antenatal care, shared care being the most common, however few women received care exclusively from GPs. The likelihood of physician involvement in care increased slightly with declining health. Recognizing these factors is essential for providing appropriate support and tailoring effective antenatal care.

## Supplementary material

10.2196/68811 Multimedia Appendix 1 The questionnaire, translated into English.

10.2196/68811 Multimedia Appendix 2 Demographic characteristics of pregnant women in Norway compared to this study.

10.2196/68811 Multimedia Appendix 3 Odds ratios with 95% CIs for good self-rated health according to perception of childhood, socioeconomic situation, and parity.

10.2196/68811 Multimedia Appendix 4 Odds ratios with 95% CIs for good self-rated mental health according to perception of childhood, current socioeconomic situation and parity.

## Abbreviations

GP: general practitioner
OR: odds ratio
SRH: self-rated health
SRMH: self-rated mental health
