# Supplementary material for: Association of Self-Rated Health in Pregnancy With Maternal Childhood Experiences, Socioeconomic Status, Parity, and Choice of Antenatal Care Providers: Cross-Sectional Study
Source: JMIR Form Res. 2025 Jun 3;9:e68811. doi: 10.2196/68811 (PMC12151455; doi:10.2196/68811)
Supplement: Multimedia Appendix 2 [file formative-v9-e68811-s002.docx]

**Multimedia Appendix 2**:

*Demographic characteristics of the pregnant women who gave birth in Norway in 2022, based on publicly available data from the Norwegian Medical Birth Registry (*<http://statistikkbank.fhi.no/mfr/>) *compared to demographic characteristics in the present study*

|  | | | **National data 2022 (n=51284)** | | | **The present study (n=1402)** | |
| --- | --- | --- | --- | --- | --- | --- | --- |
| **Variables** | | | **n** | **%** |  | **n** | **%** |
| **Age**  < 25  25-34  35-39  > 39 | | 4,118  35,296  10,296  1,770 | | 8.0  68.5  20.0  3.5 |  | **Age**  < 25 119  25-37 1,226  > 37 57 | 8.5  87.4  4.1 |
| **Parity**  Primipara  Multipara | | | 23,751  27,729 | 46.1  53.9 |  | 722  680 | 51.5  48.5 |
| **Marital status**  Married/cohabiting  Other | | | 49,010  2,470 | 95.2  4.8 |  | 1,369  33 | 97.6  2.4 |
| **Residence**  Northern Norway*  Central Norway*  Western Norway*  Eastern Norway*  Southern Norway* | 4,184  6,931  11,298  22,661  6,406 | | | 8.2  13.5  22.0  44.2  12.5 |  | 129  230  323  631  87 | 9.2  16.4  23.1  45.1  6.2 |

** Northern Norway includes counties Finnmark, Troms and Nordland. Central Norway includes counties Trøndelag and Møre og Romsdal, Western Norway includes counties Vestland and Rogaland, Eastern Norway includes counties Viken, Oslo and Innlandet, and Southern Norway includes counties Vestfold and Telemark, and Agder.*
