# Supplementary material for: Association of Self-Rated Health in Pregnancy With Maternal Childhood Experiences, Socioeconomic Status, Parity, and Choice of Antenatal Care Providers: Cross-Sectional Study
Source: JMIR Form Res. 2025 Jun 3;9:e68811. doi: 10.2196/68811 (PMC12151455; doi:10.2196/68811)
Supplement: Multimedia Appendix 3 [file formative-v9-e68811-s003.docx]

**Multimedia Appendix 3:**

Crude and adjusted odds ratios with 95% confidence intervals for good self-rated health according to perception of childhood, socioeconomic situation and parity

| **Good self-rated health** | **n**  **total** | **n**  **good SRH** | **OR**  **crude** | **95% CI** | **OR adjusted** | **95% CI** |
| --- | --- | --- | --- | --- | --- | --- |
| **Perception of childhood** | 1393 | 943 |  |  |  |  |
| Good childhood | 1076 | 755 | 1.00 | - | 1.00 | - |
| Average childhood | 178 | 114 | 0.76 | 0.54-1.06 | 0.77^1^ | 0.55-1.09 |
| Difficult childhood | 139 | 74 | 0.48 | 0.34-0.69 | 0.52^1^ | 0.36-0.76 |
|  | | | | | | |
| **Education** | 1402 | 950 |  |  |  |  |
| College/university ≥ 4 years | 564 | 423 | 1.00 | - | 1.00 | - |
| College/university <4 years | 495 | 322 | 0.62 | 0.48-0.81 | 0.62^2^ | 0.47-0.81 |
| Upper secondary school | 315 | 191 | 0.51 | 0.38-0.69 | 0.55^2^ | 0.40-0.75 |
| Lower secondary school | 28 | 14 | 0.33 | 0.16-0.72 | 0.35^2^ | 0.16-0.76 |
|  | | | | | | |
| **Current financial situation** | 1400 | 949 |  |  |  |  |
| Financial security | 1166 | 819 | 1.00 | - | 1.00 | - |
| Financial insecurity | 234 | 130 | 0.53 | 0.40-0.71 | 0.64^1^ | 0.47-0.87 |
|  | | | | | | |
| **Parity** | 1402 | 950 |  |  |  |  |
| First-time mothers | 722 | 533 | 1.00 | - | 1.00 | - |
| Given birth before | 680 | 417 | 0.56 | 0.45-0.71 | 0.53^1^ | 0.41-0.67 |

^1^ *Models are adjusted for age, trimester and education.* ^2^ *Models are adjusted for age and trimester*
