# Supplementary material for: Association of Self-Rated Health in Pregnancy With Maternal Childhood Experiences, Socioeconomic Status, Parity, and Choice of Antenatal Care Providers: Cross-Sectional Study
Source: JMIR Form Res. 2025 Jun 3;9:e68811. doi: 10.2196/68811 (PMC12151455; doi:10.2196/68811)
Supplement: Multimedia Appendix 4 [file formative-v9-e68811-s004.docx]

**Multimedia Appendix 4:**

Crude and adjusted odds ratios with 95% confidence intervals for good self-rated mental health according to perception of childhood, current socioeconomic situation and parity

| **Good self-rated mental health** | **n total** | **n**  **good SRMH** | **OR**  **crude** | **95% CI** | **OR adjusted** | **95% CI** |
| --- | --- | --- | --- | --- | --- | --- |
| **Perception of childhood** | 1393 | 1251 |  |  |  |  |
| Good childhood | 1076 | 997 | 1.00 | - | 1.00 | - |
| Average childhood | 178 | 149 | 0.58 | 0.41-0.82 | 0.57^1^ | 0.40-0.81 |
| Difficult childhood | 139 | 105 | 0.35 | 0.24-0.50 | 0.37^1^ | 0.25-0.54 |
|  | | | | | | |
| **Education** | 1402 | 1260 |  |  |  |  |
| College/university ≥ 4 years | 564 | 510 | 1.00 | - |  |  |
| College/university <4 years | 495 | 453 | 0.77 | 0.58-1.01 | 0.78^2^ | 0.58-1.03 |
| Upper secondary school | 315 | 277 | 0.51 | 0.38-0.69 | 0.55^2^ | 0.40-0.76 |
| Lower secondary school | 28 | 20 | 0.71 | 0.30-1.64 | 0.78^2^ | 0.33-1.84 |
|  | | | | | | |
| **Current financial situation** | 1400 | 1258 |  |  |  |  |
| Financial security | 1166 | 1062 | 1.00 | - | 1.00 | - |
| Financial insecurity | 234 | 196 | 0.47 | 0.35-0.64 | 0.54^1^ | 0.39-0.74 |
|  | | | | | | |
| **Parity** | 1402 | 1260 |  |  |  |  |
| First-time mothers | 722 | 650 | 1.00 | - | 1.00 | - |
| Given birth before | 680 | 610 | 0.64 | 0.50-0.81 | 0.57^1^ | 0.45-0.73 |

^1^ *Models are adjusted for age, trimester and education.* ^2^ *Models are adjusted for age and trimester*
